# Multiplex Immunofluorescence-Based Investigation of the Interventional Effect of Bisdemethoxycurcumin on Glial Cell Polarization in Radiation-Induced Brain Injury

**DOI:** 10.3390/biom16101424

**Published:** 2026-10-01

**Authors:** Tianrui Zhang, Ziying Geng, Jianhua Chen, Jianhua Li, Zongtai Han, Fangping Han, Jianguo Li

**Affiliations:** 1 Drug Safety Evaluation Center of China Institute for Radiation Protection, China, National Nuclear Corporation Key Laboratory on Radiotoxicology and Radiopharaceutical Preclinical Evaluation, Shanxi Key Laboratory of Drug Toxicology and Drug for Radiation Injury, National Atomic Energy Authority Nuclear Technology (Non-Clinical Evaluation of Radiopharmaceuticals) Research and Development Center, No. 102, Xuefu Street, Taiyuan 030006, China; 2 School of Pharmacy, Shanxi Medical University, Taiyuan 030001, China

**Keywords:** radiation-induced brain injury, bisdemethoxycurcumin, microglia, astrocyte, polarization

## Abstract

Radiation-induced brain injury (RIBI) is a severe and common complication following radiotherapy for intracranial tumors, primarily manifested as brain tissue damage, neuroinflammation, and cognitive dysfunction. Currently, clinically effective therapeutic options remain lacking, severely compromising patient prognosis and quality of life. Bisdemethoxycurcumin (BDMC) is a highly stable active derivative of curcumin with antioxidant, anti-inflammatory, and neuroprotective properties. However, the therapeutic effect and underlying mechanisms of BDMC against RIBI remain poorly defined. Objective: To investigate the neuroprotective effects of BDMC on RIBI in rats and to elucidate the molecular mechanisms that regulate glial cell polarization. Methods: Forty SPF-grade male Sprague–Dawley (SD) rats were randomly assigned to four groups (*n* = 12 per group): control, model, amifostine (AMF) and BDMC. A rat RIBI model was established by whole-brain irradiation with a single dose of 30 Gy X-rays, followed by the respective drug interventions. After 4 consecutive weeks of treatment, body weight was recorded, and brain water content and oxidative-stress indicators in brain tissue were quantified. Hematoxylin–eosin (HE) staining was performed to assess pathological injury in brain tissues. Multiplex immunohistochemistry (mIHC) was employed to quantitatively analyze the polarization states of microglia and astrocytes in the cerebral cortex, hippocampus, and thalamus. Results: Radiation-induced RIBI induced body-weight loss, cerebral edema, and cerebral oxidative-stress disturbance in rats, accompanied by neuronal edema, degeneration, and necrosis. Concurrently, microglia and astrocytes were activated in multiple brain regions, with glial polarization shifting toward the pro-inflammatory CD86^+^ microglia and C3^+^ astrocyte states and disrupting the cerebral microenvironment. BDMC intervention improved the overall condition of the rats, attenuated cerebral edema and oxidative-stress damage, and ameliorated neuronal pathological lesions. Furthermore, BDMC suppressed excessive glial activation, reversed the CD86^+^ microglia/CD206^+^ microglia and C3^+^ astrocyte/S100A10^+^ astrocyte polarization imbalance, and attenuated neuroinflammation. Conclusion: BDMC exerts significant neuroprotective effects in RIBI rats, potentially through the inhibition of cerebral oxidative stress and the maintenance of glial cell polarization homeostasis across multiple brain regions.

## 1. Introduction

With the increasing availability and clinical application of medical ionizing radiation (IR), hundreds of thousands of patients worldwide undergo radiotherapy each year for primary brain tumors and brain metastases of extracranial origin. This highlights the need to re-evaluate the literature regarding the biological effects of IR at both high and low doses, particularly its impact on the central nervous system (CNS) [1]. Radiation-induced brain injury (RIBI) develops when radiotherapy doses exceed the tolerance threshold of the CNS. IR directly damages surrounding normal brain tissue, resulting in neuronal and glial-cell apoptosis and subsequent secondary pathological responses. RIBI is primarily characterized by cerebral edema, necrosis, and demyelination, as well as functional impairments including cognitive and memory deficits [2]. Accumulated clinical evidence indicates that the incidence of radiation-induced brain injury following stereotactic radiotherapy for meningioma can reach 28–50%, which severely compromises patients’ quality of life [3]. Nevertheless, while these advanced radiotherapy techniques prolong patient survival, they also contribute to a progressive annual increase in the incidence of RIBI [4]. Therefore, in-depth investigation of the molecular and cellular mechanisms underlying RIBI and the identification of effective neuroprotective strategies have become urgent research priorities in this field.

RIBI represents a continuous, dynamic, and complex cascade process. Multiple theories have been proposed to explain its pathophysiology, including direct radiation-induced damage, activation of early-response transcription factors, signal transduction cascades, proliferative vascular and glial alterations, immune-inflammatory responses, oxidative stress, and cerebrovascular injury [5,6]. According to the temporal progression of its pathological mechanisms, radiation-induced brain injury can be divided into the following three phases [7]: (1) Acute-phase response, which is characterized primarily by direct cellular injury. Radiation induces DNA double-strand breaks in neurons and vascular endothelial cells, triggering an oxidative-stress burst and mitochondrial dysfunction, and directly initiating cellular apoptosis and necrosis. Meanwhile, rapid structural disruption and increased permeability of the blood–brain barrier (BBB) occur, accompanied by early-stage microglial activation and pro-inflammatory factor release. These events ultimately manifest as acute neurological abnormalities including cerebral edema, headache, nausea, and somnolence. (2) Subacute phase (weeks to months after irradiation), characterized by excessive glial activation and microenvironmental disturbance. Direct neuronal death is no longer predominant at this stage. Instead, sustained microglial activation and reactive astrogliosis constitute the major pathological alterations. By secreting abundant pro-inflammatory cytokines, reactive oxygen species, and toxic mediators, glial cells disrupt the homeostasis of the neural microenvironment, further aggravating synaptic injury, myelin damage, and neurological dysfunction. This phase serves as a critical transitional link between acute injury and late-stage irreversible damage. (3) Late delayed response occurring months to years after radiotherapy, characterized by chronic and irreversible tissue injury. Its major manifestations include white-matter necrosis, microvascular lesions, progressive demyelination, brain atrophy, and glial-scar formation. The underlying mechanisms involve long-term chronic inflammation, vascular occlusion, neural stem-cell exhaustion, and cellular senescence, which eventually lead to persistent cognitive decline, memory impairment, and even radiation necrosis.

Bisdemethoxycurcumin (BDMC) is a demethoxylated derivative of curcumin and is recognized as the most stable and potent curcumin-type compound [8]. Curcumin is a well-known phenolic compound extracted from *Curcuma longa* L., which possesses multiple beneficial properties, including antioxidant, anti-inflammatory, and neuroprotective activities [9]. BDMC is derived from curcumin by removal of the methoxy groups from both aromatic rings while retaining the hydroxyl groups. It exhibits higher nuclear uptake, stability, hydrophilicity, polarity, and water solubility compared with curcumin [10]. The phenolic hydroxyl groups of BDMC confer moderate free-radical quenching and chain-breaking antioxidant capacity via hydrogen atom transfer; meanwhile, the α,β-unsaturated carbonyl/enone moiety in the BDMC molecule is electrophilic and may, similar to curcumin, modify redox-sensitive cysteines such as Cys151 in Keap1, thereby attenuating Keap1–Cul3 complex-mediated ubiquitination and proteasomal degradation of Nrf2, promoting Nrf2 stabilization and nuclear translocation, further inducing canonical Nrf2 target genes including HO-1/HMOX1, NQO1 and GCLC/GCLM, and synergistically boosting endogenous antioxidant defenses such as SOD, CAT and GSH/GSSG [11,12,13]. Previous studies have demonstrated that BDMC can ameliorate learning and memory impairment induced in RIBI models by suppressing astrocytic activation and oxidative stress. Accordingly, BDMC may serve as a potential therapeutic agent for RIBI [14].

Multiplex immunohistochemistry (mIHC) differs from conventional immunohistochemistry. It enables labeling of multiple protein markers on a single tissue section [15], allowing direct visualization of the localization and relative abundance of distinct protein markers within tissues and facilitating comparative data analysis. mIHC relies on tyramide signal amplification (TSA) technology: the primary antibody specifically binds to the target antigen, and the horseradish peroxidase (HRP)-conjugated secondary antibody is recruited to the antigen site. In the presence of hydrogen peroxide, HRP activates fluorophore-labeled tyramide substrates. The activated tyramine free radicals form irreversible covalent bonds with tyrosine residues of adjacent tissue proteins, permanently anchoring fluorescent signals in situ at the antigen location. The primary antibody-secondary antibody complex can then be eluted while retaining the covalently deposited fluorescent signals, enabling multiple rounds of sequential staining [16]. mIHC overcomes the limitation imposed by the species origin of primary and secondary antibodies, enabling the use of multiple antibodies raised in the same host species for staining [17]. At present, multiplex immunohistochemistry is mostly applied in tumor diagnosis. For studies focusing on central nervous system glial cells, immunofluorescence double staining remains the mainstream technique. However, this method cannot compare the polarization degrees of microglia and astrocytes on a single tissue section, and there are few reports regarding the application of multiplex immunohistochemistry in the central nervous system. Therefore, the introduction of the mIHC technique is of great significance for exploring the mechanisms underlying radiation-induced brain injury. In view of the above-mentioned limitations, this study adopted mIHC to investigate the activation status of microglia and astrocytes in RIBI. We aim to explore the pathogenesis of neuroinflammation in RIBI using this novel histopathological technique, so as to provide new therapeutic strategies and potential targets.

## 2. Materials and Methods

### 2.1. Experimental Animals and Grouping

A total of 48 specific-pathogen-free (SPF) male Sprague–Dawley (SD) rats aged 7 weeks were used in this experiment. The animals were purchased from Beijing Vital River Laboratory Animal Technology Co., Ltd. (production license: SCXK (Jing) 2021-0011, Beijing, China). All rats were raised in a barrier environment with a laboratory animal use license (SYXK (Jin) 2025-0002), and all animal husbandry procedures were performed by qualified personnel. All animal experiments were approved by the Institutional Animal Care and Use Committee of China Institute for Radiation Protection (approval number: CIRP-IACUC-(R)2026016, approval date: 19 March 2026). The animal study was performed in an AAALAC International-accredited facility, in accordance with the Guide for the Care and Use of Laboratory Animals and the ARRIVE guidelines.

The temperature and humidity of the animal room were automatically monitored and recorded by an environmental monitoring platform, with a controlled temperature range of 20–26 °C and humidity of 30–70%, and a 12 h light/dark cycle. During the entire experiment, the rats were housed in standard breeding cages with five animals per cage. The cages were kept clean and dry, and cage changing was performed twice a week. The ventilation frequency of the experimental room was maintained at no less than 15 air changes per hour during experimental operations and no less than 10 air changes per hour during non-working hours. The laboratory was cleaned daily after operation, and disinfection with different types of disinfectants was performed alternately every week.

The 48 SD rats were randomly divided into four groups (12 rats per group) according to body weight using a random-number table: control group, model group, positive-drug group (amifostine, AMF group), and BDMC group.

### 2.2. Main Reagents

Bisdemethoxycurcumin (purity ≥ 98%) was purchased from Shanghai Yuanye Bio-Technology Co., Ltd. (Batch No. JS243326, Shanghai, China). Dimethyl sulfoxide (DMSO) was purchased from Shanghai Yuanye Bio-Technology Co., Ltd. (Batch No. 20250901503, Shanghai, China). Zoletil^®^ 50 was obtained from Beijing Boerxi Technology Co., Ltd. (Batch No. 9T1WA, Beijing, China). Superoxide dismutase (SOD) assay kit was obtained from Nanjing Jiancheng Bioengineering Institute (Batch No. A001-1-2, Jiangsu, China). Catalase (CAT) assay kit was obtained from Nanjing Jiancheng Bioengineering Institute (Batch No. A007-1-1, Jiangsu, China). Glutathione peroxidase (GSH-Px) assay kit was obtained from Nanjing Jiancheng Bioengineering Institute (Batch No. A005-1-2, Jiangsu, China). Malondialdehyde (MDA) assay kit was obtained from Nanjing Jiancheng Bioengineering Institute (Batch No. A003-1-2, Jiangsu, China). TSA Fluorescence Kits were obtained from Panovue (Cat. No. 10021001060, Beijing, China). The PANO 7-plex IHC kit was purchased from Panovue (Cat. No. 10217100100, Beijing, China). IBA1 antibody (Batch No. R382207), GFAP antibody (Batch No. R22776), C3 antibody (Batch No. ab200999), S100A10 antibody (Batch No. bs-8503R), CD86 antibody (Batch No. bs-1035R), CD206 antibody (Batch No. HA722892). Amifostine for injection was purchased from Merro Pharmaceutical Co., Ltd. (Batch No. 3201202, Dalian, China).

### 2.3. Main Instruments and Software

Linear accelerator (Elekta Limited, Crawley, UK, 6 MeV); Microplate reader (Multiskan Go, Thermo Fisher Scientific, Vantaa, Finland); Olympus VS200 whole-slide scanning platform (Olympus VS200 MTL, Tokyo, Japan); Optical microscope (Nikon, Eclipse Ei, Tokyo, Japan). QuPath software (v0.5.1) and image J (1.49v) for image analysis and R Script (Version 4.4.2) for data analysis.

### 2.4. Administration Regimens

In this experiment, both the BDMC-treated group and AMF-treated group received drug administration via intraperitoneal (i.p.) injection. BDMC was dissolved in distilled water containing 5% dimethyl sulfoxide (DMSO) at a dosage of 100 mg/kg with an injection volume of 10 mL/kg. [14] The drug was administered once daily for four consecutive weeks. AMF was dissolved in normal saline at a dosage of 60 mg/kg with an injection volume of 10 mL/kg, and a single injection was performed only 30 min prior to radiation exposure.

### 2.5. Establishment of the RIBI Model

All rats were weighed prior to irradiation. Experimental animals were anesthetized via subcutaneous neck injection of Zoletil^®^ 50 (30 mg/kg). Rats in the Model, AMF and BDMC groups received whole-brain X-ray irradiation at a single dose of 30 Gy, with a dose rate of 300 MU/min, source-to-skin distance of 100 cm, and irradiation rate of 3 Gy/min. Rats in the Control group were placed in the irradiation device, while a 12 cm thick lead block was used to completely shield the radiation source, so as to exclude the stress effects induced by the irradiation environment.

### 2.6. Cage-Side Observation

All animals underwent daily cage-side observations. Observations included general appearance (fur condition and alopecia), physical signs (body temperature and spontaneous tremors), behavioral activities (locomotion and abnormal postures), glandular secretions (oral secretions), respiratory status (tachypnea), fecal characteristics (fecal consistency), and mortality.

### 2.7. Body Weight Measurement

Body weights of all rats were measured throughout the experimental period. Rats were weighed 1 day before irradiation, 1 day after irradiation, and once weekly following radiation exposure.

### 2.8. Brain Edema Assessment

Rats were sacrificed for brain water content measurement at 4 weeks post-irradiation. Brain tissues were harvested and weighed to obtain the wet weight (W). Subsequently, the tissues were oven-dried for 72 h and weighed to record the constant dry weight (D). The brain edema rate (%) was calculated using the following formula: Brain edema rate (%) = (W − D)/W × 100%.

### 2.9. Biochemical Assays

Rats were sacrificed at 4 weeks post-irradiation. Brain tissues were collected and homogenized in pre-cooled PBS at a tissue-to-buffer ratio of 1:9. The homogenate was centrifuged at 3000 rpm for 10 min at 4 °C, and the supernatant was harvested. The following indicators were measured according to the manufacturer’s instructions for biochemical assay kits: superoxide dismutase (SOD), malondialdehyde (MDA), catalase (CAT), and glutathione peroxidase (GSH-Px).

### 2.10. Histopathological Examination

SD rats were sacrificed at 4 weeks post-irradiation. Brain tissues were collected and fixed in 10% formalin solution at room temperature (20–25 °C) for 72 h. Following gradient ethanol dehydration and xylene clearing, tissues were embedded in paraffin routinely. Sections were cut at 4 μm thickness and stained with hematoxylin and eosin (HE). Finally, the histomorphological alterations of brain tissues were observed under a light microscope.

### 2.11. Multiplex Fluorescence Immunohistochemistry

After brain tissue embedding and sectioning, paraffin sections were de-waxed and immersed in antigen retrieval buffer. The sections were boiled at high microwave power, followed by low-power maintenance for 15 min (supplementary buffer was added during the procedure to prevent section drying). Subsequently, the sections were taken out and allowed to cool naturally to room temperature.

Blocking buffer was applied, and sections were incubated with shaking under humidified conditions at room temperature for 10 min. After removal of blocking buffer, primary antibody working solution was added, and sections were incubated with shaking under humidified conditions at room temperature for 1 h, followed by washing three times in 1 × TBST buffer for 3 min each. HRP-conjugated secondary antibody working solution was applied directly, and sections were incubated under humidified conditions at room temperature for 10 min, then washed three times in 1 × TBST buffer for 3 min each.

Then, 100 μL of 1 × dye working solution was added. Sections were incubated with shaking under humidified conditions at room temperature for 10 min and washed three times in 1 × TBST buffer for 3 min each. After microwave-mediated antigen retrieval, sections were cooled naturally to room temperature, rinsed once with sterile water, and immersed in 1 × TBST buffer for 2 min to complete one round of staining. This staining cycle was repeated five times.

Finally, DAPI working solution was applied for incubation under humidified conditions at room temperature. Sections were washed in 1 × TBST buffer for 3 min at room temperature and rinsed with sterile water for 2 min. When slides were nearly dry, anti-fade mounting medium was applied, and coverslips were placed for mounting. Whole-slide multicolor fluorescence scanning was performed using an Olympus VS200 MTL (Olympus, Tokyo, Japan) with an Olympus UPLXAPO20X (Olympus, Tokyo, Japan) objective lens. Quantitative pathological analysis of multicolor fluorescence images across the entire slide was carried out using QuPath software.

### 2.12. Image Analysis

In this study, Image J software was used for fluorescence analysis of single-channel fluorescent images, while QuPath combined with R scripts was applied for cell counting of multichannel fluorescent images. Regions of interest (ROIs) were uniformly sampled from the entire parietal cerebral cortex, hippocampus (CA1, CA2, CA3 subfields and dentate gyrus), and ventral posterolateral nucleus (VPL) of the thalamus across all animals and experimental groups.

### 2.13. Statistical Analysis

Statistical analyses were performed using SAS 8.2 software. The Shapiro–Wilk test was used to assess the normality of data distribution, and Levene’s test was used to evaluate homogeneity of variances. Data with a normal distribution were expressed as mean ± standard deviation (SD). For normally distributed data, an independent-samples t-test was used for comparisons between two groups, and one-way analysis of variance (ANOVA) was applied for comparisons among three or more groups. The least-significant difference (LSD) method was used for post hoc pairwise comparisons. Non-parametric tests were adopted when data did not conform to a normal distribution. The significance level was set at *p* < 0.05.

## 3. Results

### 3.1. General Observations of Rats

We also performed general observations throughout the experiment to evaluate the effects of BDMC on the overall physical condition of irradiated rats. Before irradiation, all animals exhibited normal food intake, alert responses, smooth fur and good activity, with no skin trauma, tail deformity, or secretions around the oronasal and perianal regions. Within the first week after irradiation, all irradiated animals showed transient lethargy, hypoactivity and reduced appetite. No obvious gross abnormalities were observed in any group after this period, which is consistent with the typical manifestations following cranial radiation injury. No animal death occurred during the entire experiment. The primary endpoints of this study were radiation-induced brain pathological injury, oxidative stress biomarkers and radioprotective effects. General animal condition was only monitored for routine animal welfare surveillance to exclude unexpected deaths. We did not adopt a standardized quantitative scoring system, as these clinical signs served merely as descriptive screening indicators rather than primary outcomes for hypothesis testing. No obvious intergroup differences in general appearance and activity were observed throughout most of the observation period, except during the first week after irradiation.

### 3.2. Intervention Effect of BDMC on Body Weight in Rats

**Figure 1:**
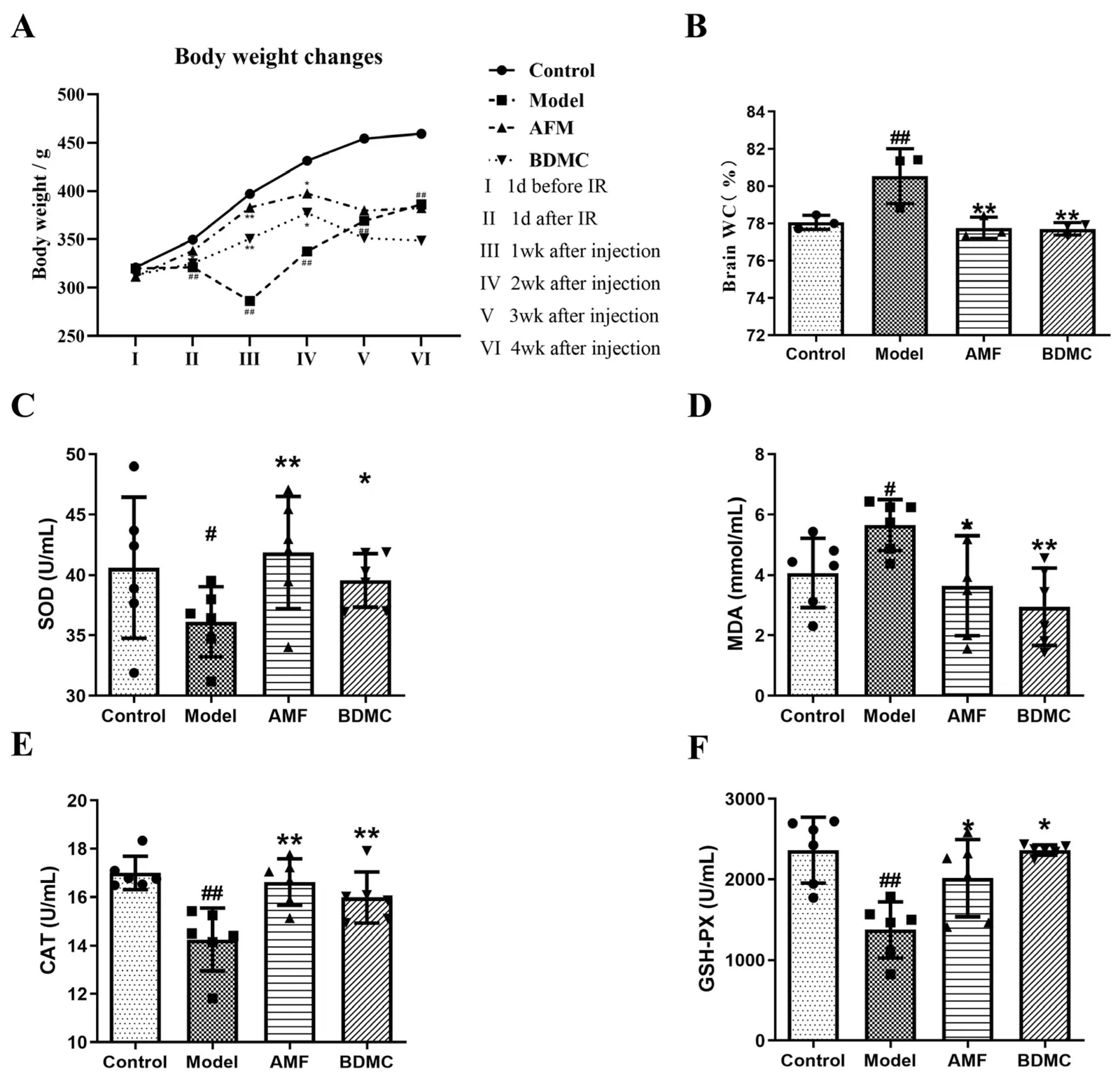
Intervention effect of BDMC on RIBI rats. (**A**) Body weight changes in rats in each group (*n* = 12); (**B**) changes in brain water content of rats in each group (*n* = 3); (**C**–**F**) alterations in cerebral oxidative stress levels in each group (*n* = 6). Data in this figure were analyzed by one-way ANOVA with an LSD post hoc test. The significance level was set at 0.05. * *p* < 0.05, ** *p* < 0.01 vs. Model group; # *p* < 0.05, ## *p* < 0.01 vs. Control group.

As shown in Figure 1A, the results showed that there was no significant difference in body weight among all groups one day before irradiation (*p* > 0.05). The Model group exhibited a marked sharp drop in body weight at week 1 after irradiation (*p* < 0.01). This acute weight loss was mainly induced by transient acute radiation stress, accompanied by temporary inappetence and lethargy. After this acute phase, food intake gradually recovered, and the body weight of the Model group rebounded steadily. The AMF and BDMC treatment groups maintained continuous weight gain throughout most of the observation period and did not display the prominent weight loss observed in the Model group (*p* < 0.01). At 3–4 weeks post-irradiation, there was no significant difference in body weight between the Model group and the two treatment groups. However, the body weights of the Model, AMF and BDMC groups were all lower than those of the Control group, indicating that irradiation caused persistent mild growth retardation [18]. Such sustained growth retardation may be related to neuroendocrine disturbance and low-grade chronic neuroinflammation induced by brain irradiation, even after the resolution of acute radiation sickness [19].

### 3.3. Intervention Effect of BDMC on Brain Water Content in Rats

In the present study, changes in brain water content of rats were determined. Under radiation exposure, obvious cerebral edema occurred in the Model group, with the brain water content reaching 81%, which was significantly different from that of the Control group. After treatment with AMF and BDMC, the brain water content of rats was restored to the normal level of 78%, comparable to the Control group. See Figure 1B for details.

### 3.4. Intervention Effect of BDMC on Oxidative Stress in Rat Brain Tissue

To explore the effect of BDMC on oxidative stress levels in rats with RIBI, the contents of superoxide dismutase (SOD), malondialdehyde (MDA), catalase (CAT), and glutathione peroxidase (GSH-Px) in brain tissue were determined for each group. The results demonstrated that compared with the Control group, the RIBI Model group exhibited significantly decreased levels of SOD, CAT, and GSH-Px, alongside markedly elevated MDA content, with statistically significant inter-group differences (*p* < 0.05). Compared with the Model group, the AMF group and BDMC group showed obvious recovery in the activities of SOD, CAT and GSH-Px, accompanied by a significant reduction in MDA content (*p* < 0.05). Meanwhile, no significant differences in all oxidative-stress-related indices were observed between the AMF group, BDMC group and the Control group (*p* > 0.05). These findings confirm that BDMC can effectively reverse oxidative-stress disturbance induced by radiation-induced brain injury and improve the oxidative-stress status of rats. See Figure 1C–F for details.

### 3.5. Intervention Effect of BDMC on the Cerebral Cortex, Hippocampus and Thalamus in RIBI Rats

To observe the protective effect of BDMC on brain structure in RIBI rats, brain tissue sections were subjected to hematoxylin–eosin (HE) staining. Microscopic observation revealed that:

Cerebral cortex: In the Control group, the cerebral cortex showed well-defined layers and evenly distributed neurons. Neurons possessed abundant cytoplasm and distinct nucleoli. No pathological abnormalities such as edema, degeneration or necrosis were observed. Severe pathological injuries were found in the cerebral cortex of the RIBI Model group. Microscopically, neuronal edema as well as vacuolated and translucent cytoplasm could be seen. A large number of neurons exhibited pyknosis with shrunken and dark-stained nuclei, and some neurons presented soma fragmentation, necrosis and shedding. Compared with the Model group, neuronal morphology was markedly restored in the AMF group and BDMC-intervention group. Obvious cellular edema was absent; only a small number of neurons with mild pyknosis were scattered, and extensive cell necrosis was not detected.

Hippocampus: In the Control group, hippocampal neurons were densely and regularly arranged with intact cellular morphology. Neurons possessed abundant cytoplasm and distinct nucleoli. No interstitial edema, degeneration or necrosis was observed, and the laminated structure of the hippocampus remained intact. In the Model group, the CA1 and CA2 subregions of the hippocampus suffered the most severe radiation-induced injury, showing disorganized neuronal arrangement and partial neuronal loss. Neuronal edema and multiple severe pyknosis were observed, accompanied by focal neuronal necrosis and severe disruption of normal cellular architecture. Compared with the Model group, neuronal damage in the AMF and BDMC-intervention groups was markedly alleviated, with reduced cell loss. Edema and vacuolar degeneration were significantly mitigated. Only scattered neurons with mild pyknosis could be seen, without massive necrosis, and the hippocampal lamination was largely preserved.

**Figure 2:**
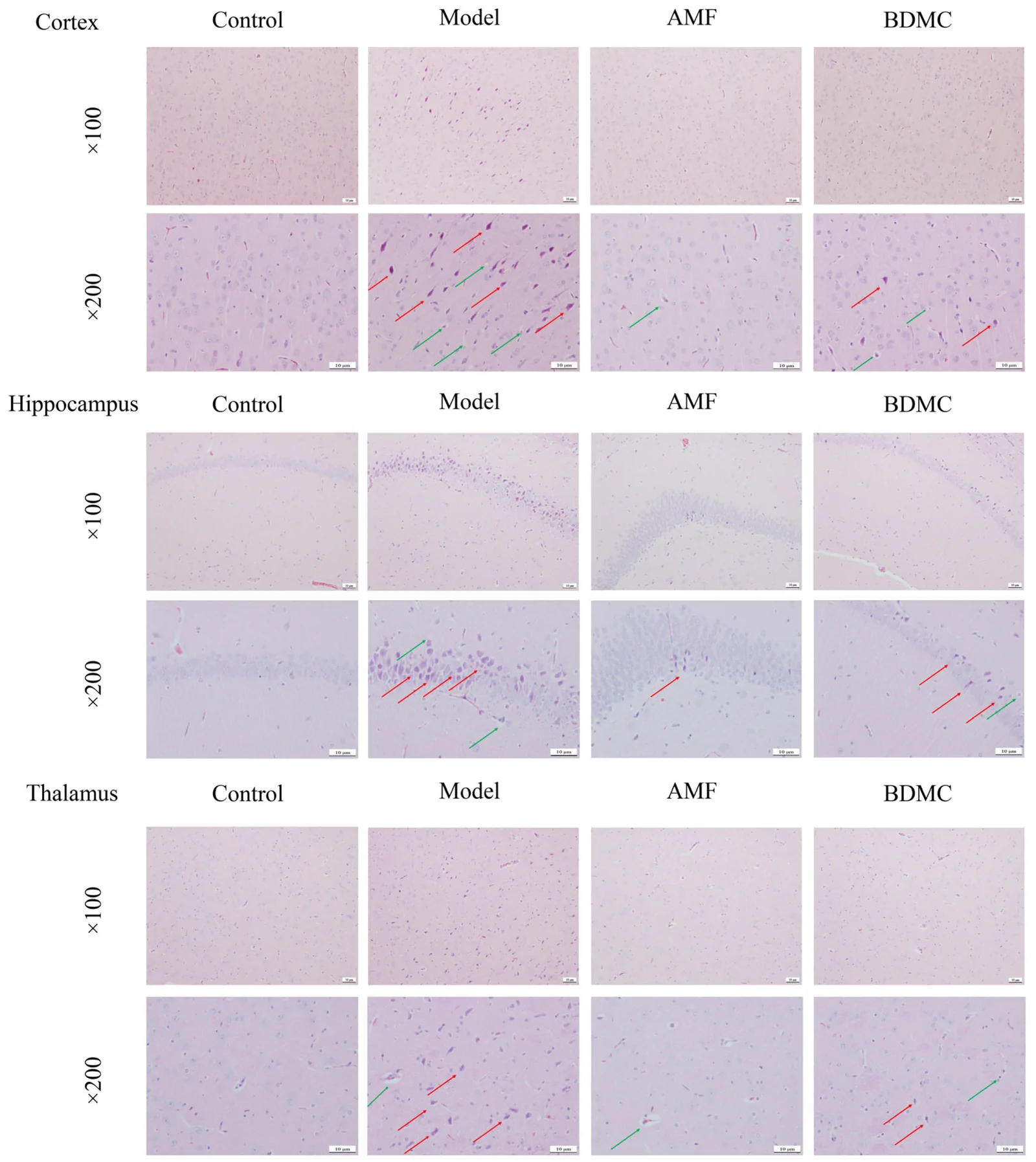
Effects of BDMC on neurons in the cerebral cortex, hippocampus and thalamus of RIBI rats. *n* = 3, HE staining, ×200. Scale bar = 10 μm. Representative images were obtained from the parietal cerebral cortex (layers III–V), hippocampal CA1 subfield, and ventral posterolateral nucleus (VPL) of the thalamus. Red arrows indicate neuronal degeneration, and green arrows indicate edema.

Thalamus: In the Control group, thalamic neurons were evenly distributed and uniform in size, with normal nuclear morphology and plump cytoplasm. The interstitium was compact and free of edema; no vacuolar degeneration or necrosis was detected, and the gray-matter structure remained intact. Moderate radiation-induced injury occurred in the thalamus of the Model group: neurons presented widespread edema with vacuolated and lightly stained cytoplasm, and some neurons showed deeply stained pyknosis. Loose interstitial edema and dilated perivascular spaces were observed, accompanied by focal mild neuronal lytic necrosis and disorganized neuronal arrangement. Compared with the Model group, thalamic injury was significantly alleviated in the AMF and BDMC-intervention groups. Neuronal edema and vacuolar alterations were largely diminished, pyknotic neurons were rare, and no focal confluent necrosis was found. Neuronal arrangement returned to uniformity, interstitial edema subsided, and the integrity of gray-matter structure was obviously improved. Collectively, these findings demonstrate that BDMC can effectively ameliorate neuronal degeneration, necrosis and edema caused by radiation-induced brain injury, and markedly repair histopathological damage of neural tissues. See Figure 2 for details.

Meanwhile, we performed semi-quantitative analysis of pathological lesions in three brain regions, including the hippocampus, cerebral cortex and thalamus. The scoring criteria were adopted according to internationally accepted pathological methods: 0 = no abnormality, 1 = minimal lesion, 2 = mild lesion, 3 = moderate lesion, 4 = severe lesion [20]. The scores in Table 1 cover two pathological features, neuronal pyknosis and edema, with a maximum total score of 8. Detailed information is presented in Table 1.

### 3.6. Intervention Effect of BDMC on Glial Cells in the CNS of RIBI Rats

**Figure 3:**
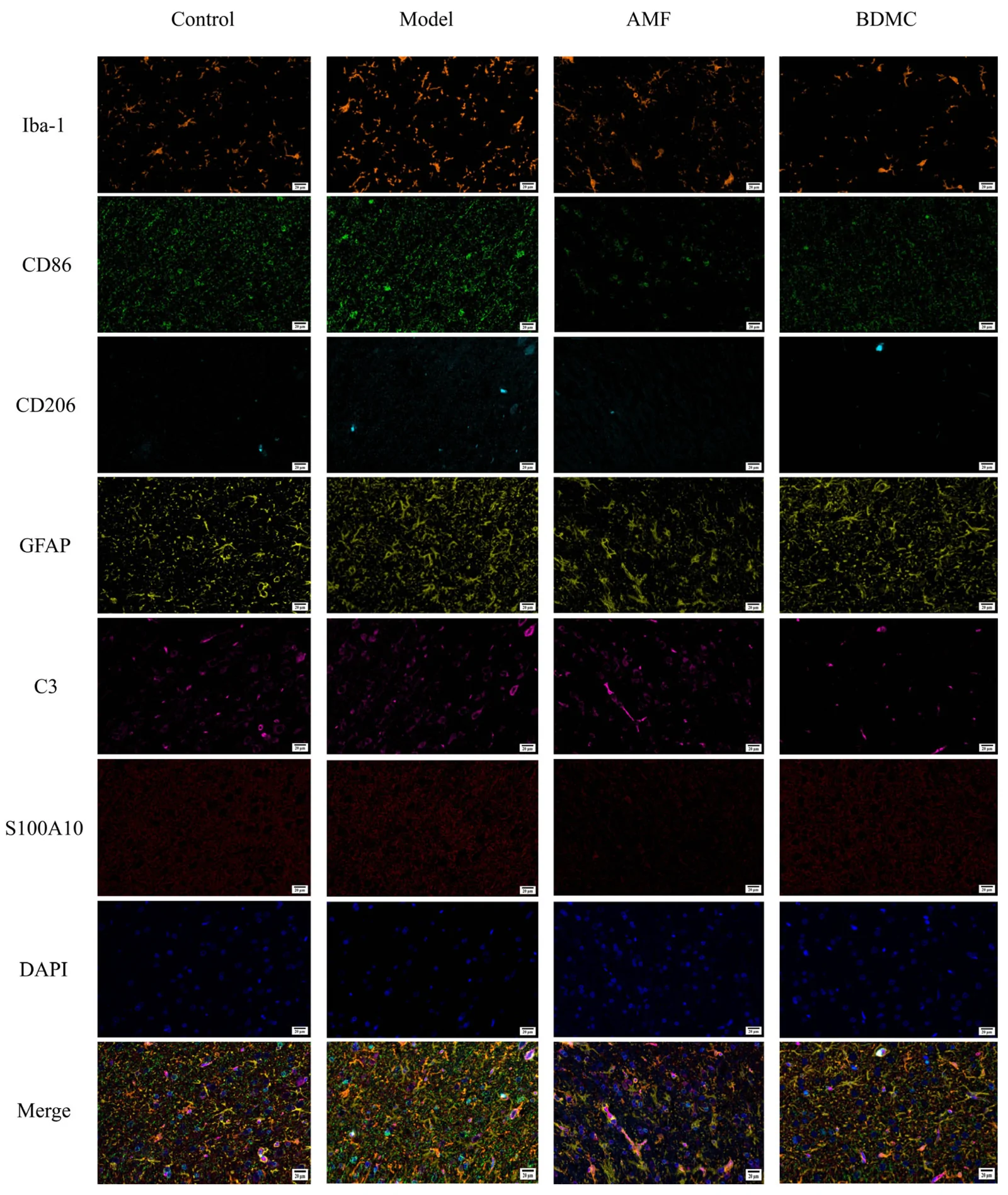
Expression of microglia and glial cells in the cerebral cortex of RIBI rats (*n* = 3). Scale bar = 20 μm. Representative images were obtained from the parietal cerebral cortex (layers III–V).

**Figure 4:**
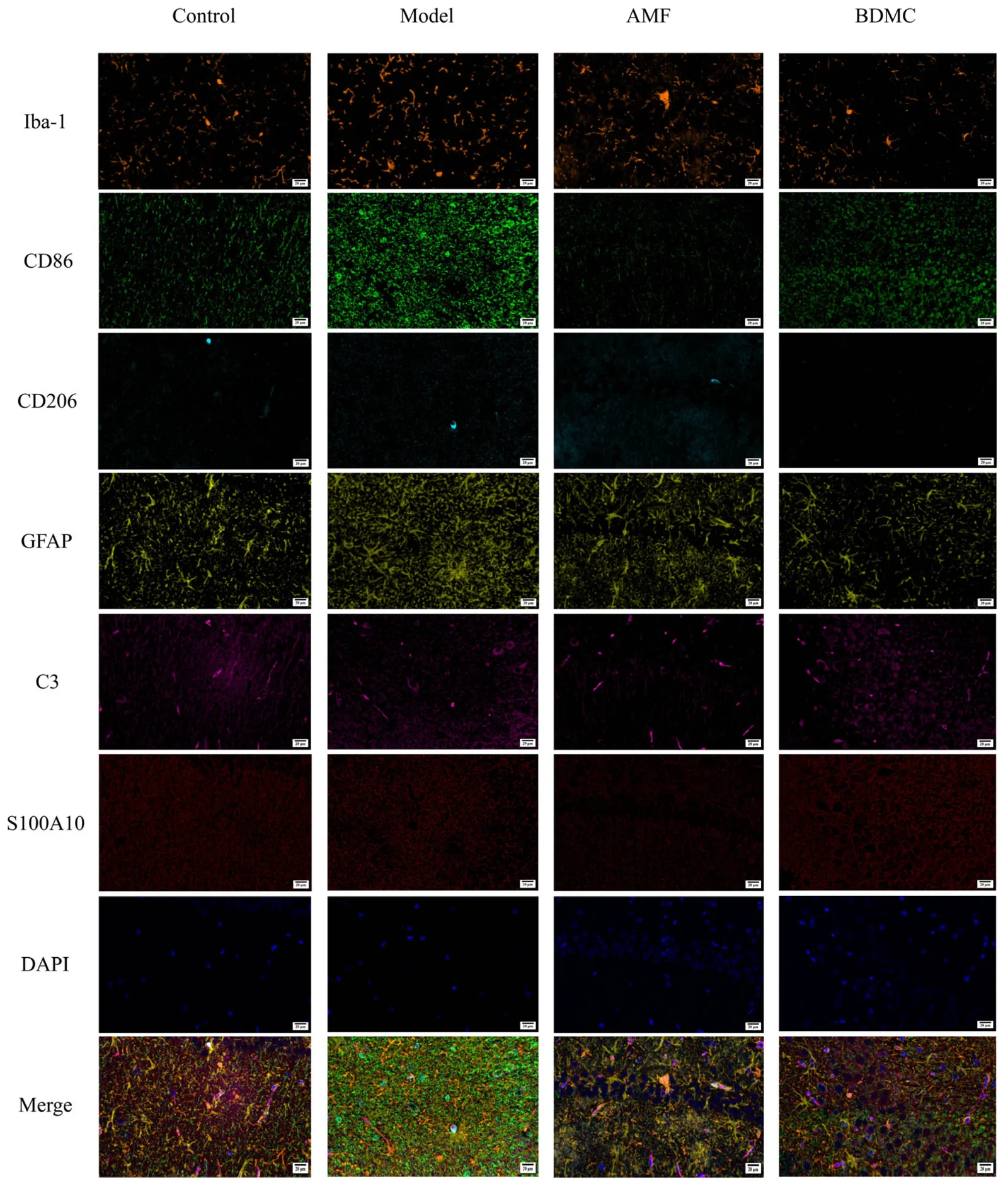
Expression of microglia and glial cells in the hippocampus of RIBI rats (*n* = 3). Scale bar = 20 μm. Representative images were obtained from the hippocampal CA1 astrocyte subfield.

**Figure 5:**
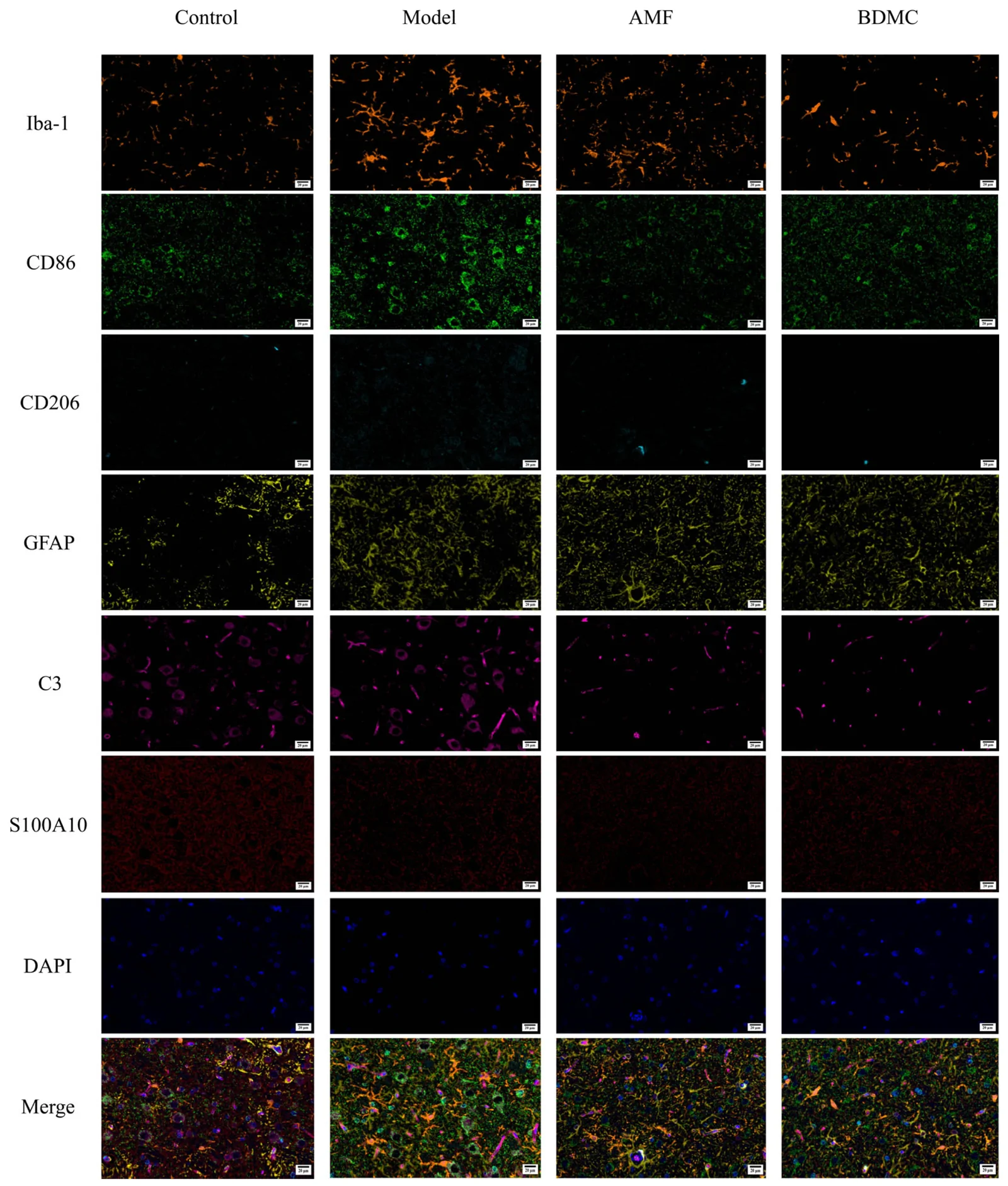
Expression of microglia and glial cells in the thalamus of RIBI rats (*n* = 3). Scale bar = 20 μm. Representative images were obtained from the VPL of the thalamus.

**Figure 6:**
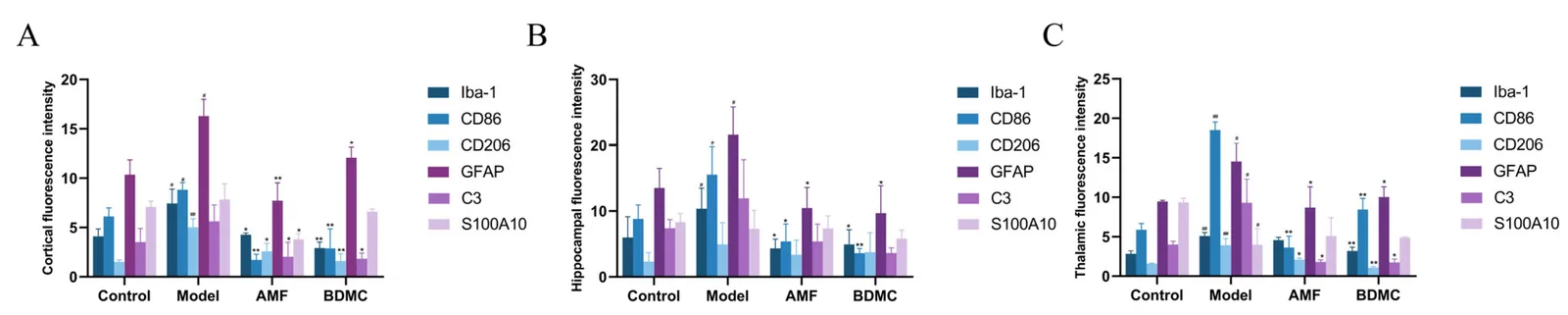
Quantitative analysis of fluorescence intensity from single-channel immunofluorescence. (**A**) Quantitative statistics of fluorescence intensity in the cerebral cortex; (**B**) Quantitative statistics of fluorescence intensity in the hippocampus; (**C**) Quantitative statistics of fluorescence intensity in the thalamus. Data in this figure were analyzed by one-way ANOVA with an LSD post hoc test. The significance level was set at 0.05. * *p* < 0.05, ** *p* < 0.01 vs. Model group; # *p* < 0.05, ## *p* < 0.01 vs. Control group.

Rat brain tissue sections were stained using mIHC technology. In immunofluorescence images, orange fluorescence indicated the microglial marker IBA1; green fluorescence represented the CD86^+^ microglia-state marker CD86; sky-blue fluorescence corresponded to the CD206^+^ microglia-state marker CD206; yellow fluorescence denoted the astrocyte marker GFAP; pink fluorescence indicated the C3^+^ astrocyte-state marker C3; red fluorescence represented the S100A10^+^ astrocyte-state marker S100A10; and blue DAPI fluorescence marked cell nuclei. See Figure 3, Figure 4, Figure 5 and Figure 6 for details.

In the cerebral cortex, hippocampus, and thalamus, significantly increased expression of Iba-1 and GFAP was observed in the Model Group compared with the Control Group (*p* < 0.05), indicating activation and up-regulation of microglia and astrocytes in major brain regions of rats following irradiation. In the cerebral cortex, the Model Group exhibited significant differences in CD86 and CD206 relative to the Control Group (*p* < 0.05). Although the mean values of C3 and S100A10 were higher in the Model Group, no significant difference was detected. In the hippocampus, CD86 showed a significant difference in the Model Group versus the Control Group (*p* < 0.05). In the thalamus, significant differences in CD86, CD206, C3, and S100A10 were found in the Model Group compared with the Control Group (*p* < 0.05).

In the cerebral cortex, hippocampus, and thalamus, significantly down-regulated expression of Iba-1 and GFAP was observed in the BDMC Group compared with the Model Group (*p* < 0.05), indicating that intraperitoneal injection of BDMC suppressed the activation of microglia and astrocytes in major brain regions of rats. In the cerebral cortex, the BDMC Group exhibited significant differences in CD86, CD206, and C3 relative to the Model Group (*p* < 0.05). Although the mean value of S100A10 was lower in the BDMC Group, no significant difference was detected. In the hippocampus, CD86 showed a significant difference in the BDMC Group versus the Model Group (*p* < 0.01). In the thalamus, significant differences in CD86, CD206, and C3 were found in the BDMC Group compared with the Model Group (*p* < 0.05).

### 3.7. Regulatory Effects of BDMC on CD86^+^ Microglia/CD206^+^ Microglia and C3^+^ Astrocyte/S100A10^+^ Astrocyte

We detected the expression of CD86^+^ microglia (Iba-1/CD86 double-positive), CD206^+^ microglia (Iba-1/CD206 double-positive), C3^+^ astrocytes (GFAP/C3 double-positive), and S100A10^+^ astrocytes (GFAP/S100A10 double-positive) in the cerebral cortex, hippocampus, and thalamus of rats, respectively.

#### 3.7.1. Microglial Polarization

**Figure 7:**
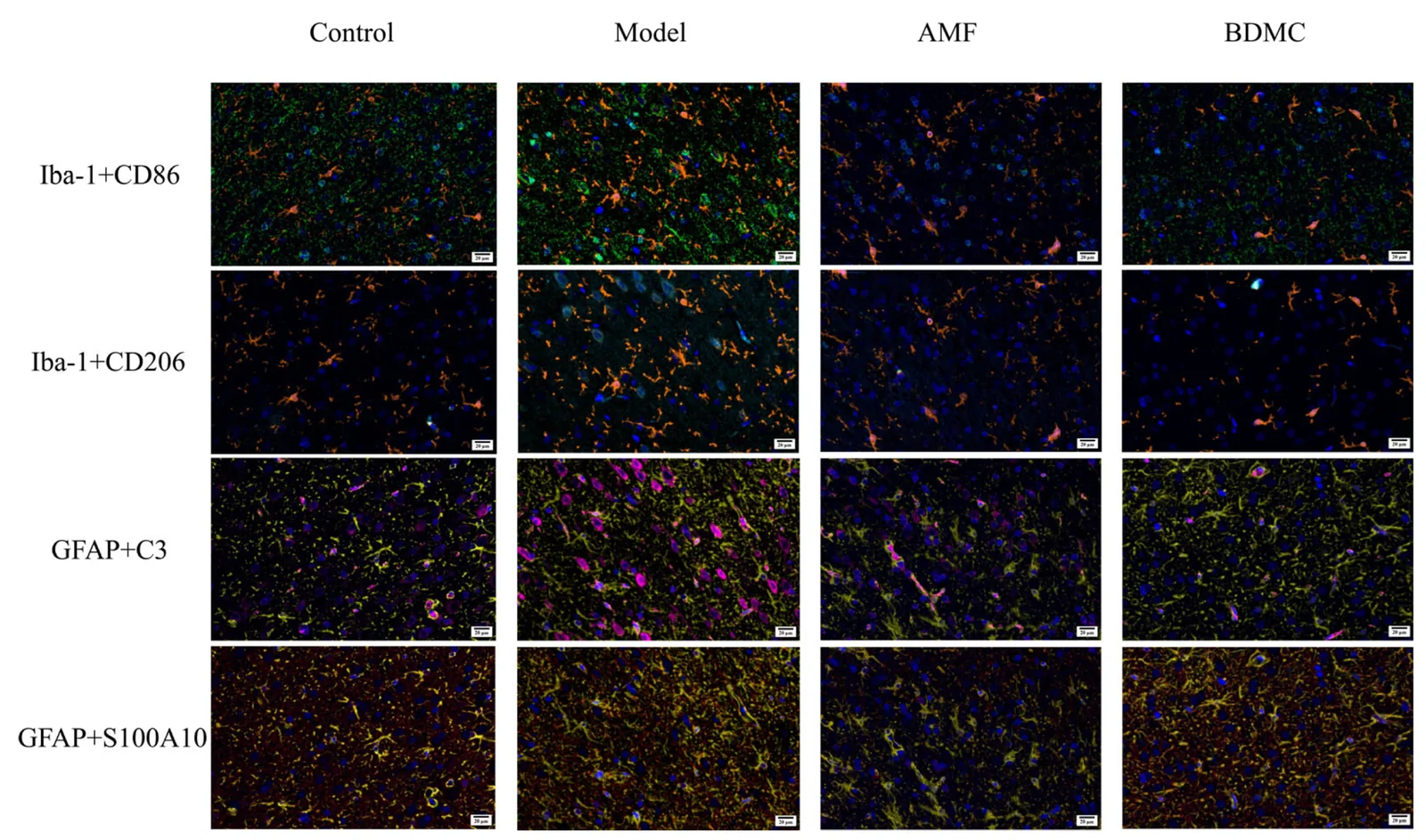
Expression of CD86^+^ microglia/CD206^+^ microglia and C3^+^ astrocyte/S100A10^+^ astrocyte in the cerebral cortex of RIBI rats (*n* = 3). Scale bar = 20 μm. Representative images were obtained from the parietal cerebral cortex (layers III–V).

**Figure 8:**
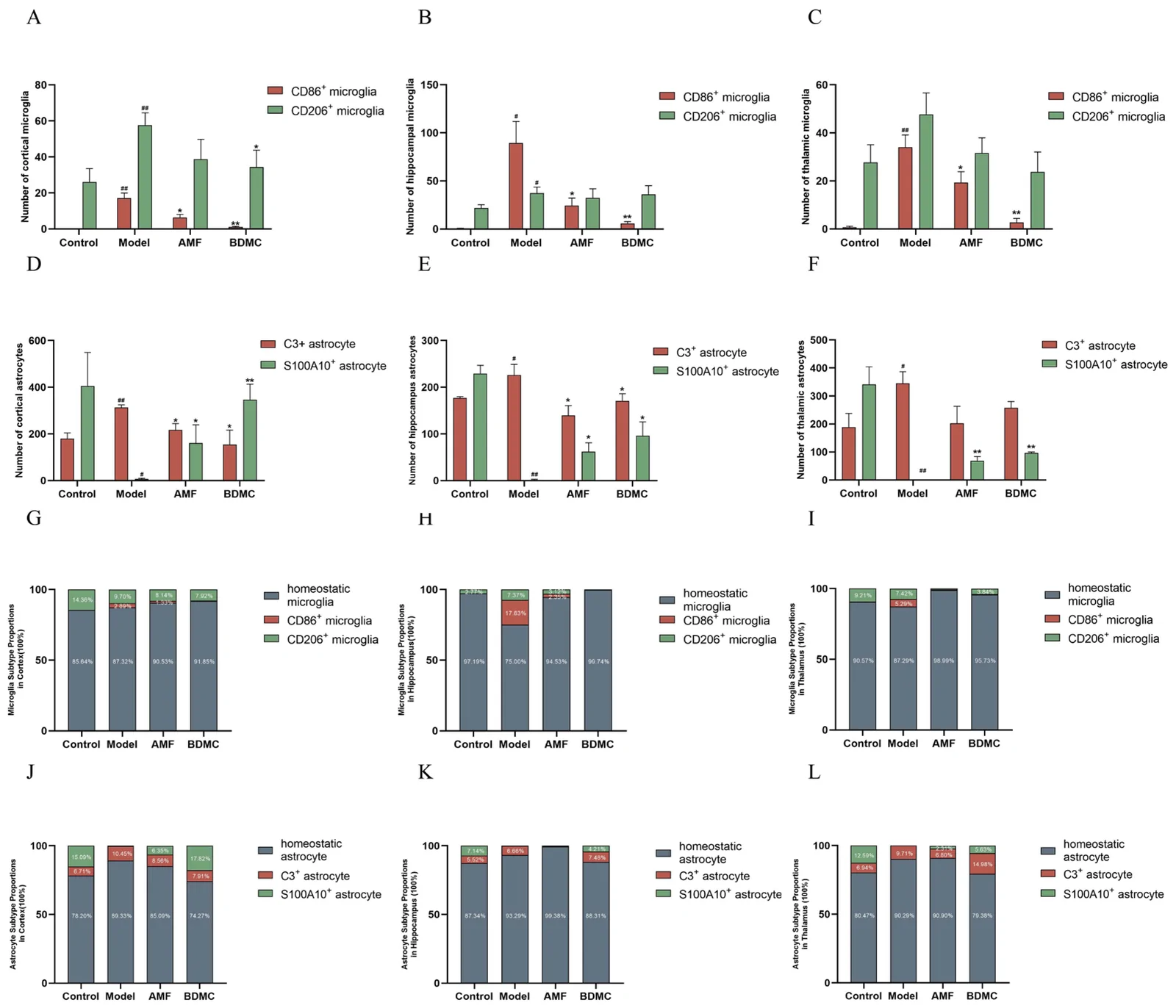
Quantitative analysis of the number and proportion of CD86^+^ microglia/CD206^+^ microglia and C3^+^ astrocyte/S100A10^+^ astrocyte. (**A**) Number of CD86^+^ microglia and CD206^+^ microglia in the cerebral cortex; (**B**) Number of CD86^+^ microglia and CD206^+^ microglia in the hippocampus; (**C**) Number of CD86^+^ microglia and CD206^+^ microglia in the thalamus; (**D**) Number of C3^+^ astrocyte and S100A10^+^ astrocyte in the cerebral cortex; (**E**) Number of C3^+^ astrocyte and S100A10^+^ astrocyte in the hippocampus; (**F**) Number of C3^+^ astrocyte and S100A10^+^ astrocyte in the thalamus; (**G**) Proportion of homeostatic microglia,CD86^+^ microglia, and CD206^+^ microglia in the cerebral cortex; (**H**) Proportion of homeostatic microglia, CD86^+^ microglia, and CD206^+^ microglia in the hippocampus; (**I**) Proportion of homeostatic microglia, CD86^+^ microglia, and CD206^+^ microglia in the thalamus; (**J**) Proportion of homeostatic astrocyte,C3^+^ astrocyte, and S100A10^+^ astrocyte in the cerebral cortex; (**K**) Proportion of homeostatic astrocyte, C3^+^ astrocyte, and S100A10^+^ astrocyte in the hippocampus; (**L**) Proportion of homeostatic astrocyte, C3^+^ astrocyte, and S100A10^+^ astrocyte in the thalamus. Data in this figure were analyzed by one-way ANOVA with an LSD post hoc test. The significance level was set at 0.05. * *p* < 0.05, ** *p* < 0.01 vs. Model group; # *p* < 0.05, ## *p* < 0.01 vs. Control group.

In the cerebral cortex, the number of CD86^+^ microglia was significantly increased in the Model Group compared with the Control Group (*p* < 0.01), accompanied by a marked elevation in CD206^+^ microglia (*p* < 0.01). Following AMF intervention, the count of CD86^+^ microglia was significantly decreased (*p* < 0.05), with a concomitant reduction in CD206^+^ microglia. In the BDMC Group, CD86^+^ microglia were significantly down-regulated (*p* < 0.01); the number of CD206^+^ microglia declined relative to the Model Group yet remained higher than that in the Control Group. These findings indicate that irradiation triggers bidirectional polarization of microglia toward both CD86^+^ microglia and CD206^+^ microglia states in the cerebral cortex, whereas AMF and BDMC are capable of suppressing CD86^+^ microglia polarization. See Figure 7 and Figure 8A for details.

**Figure 9:**
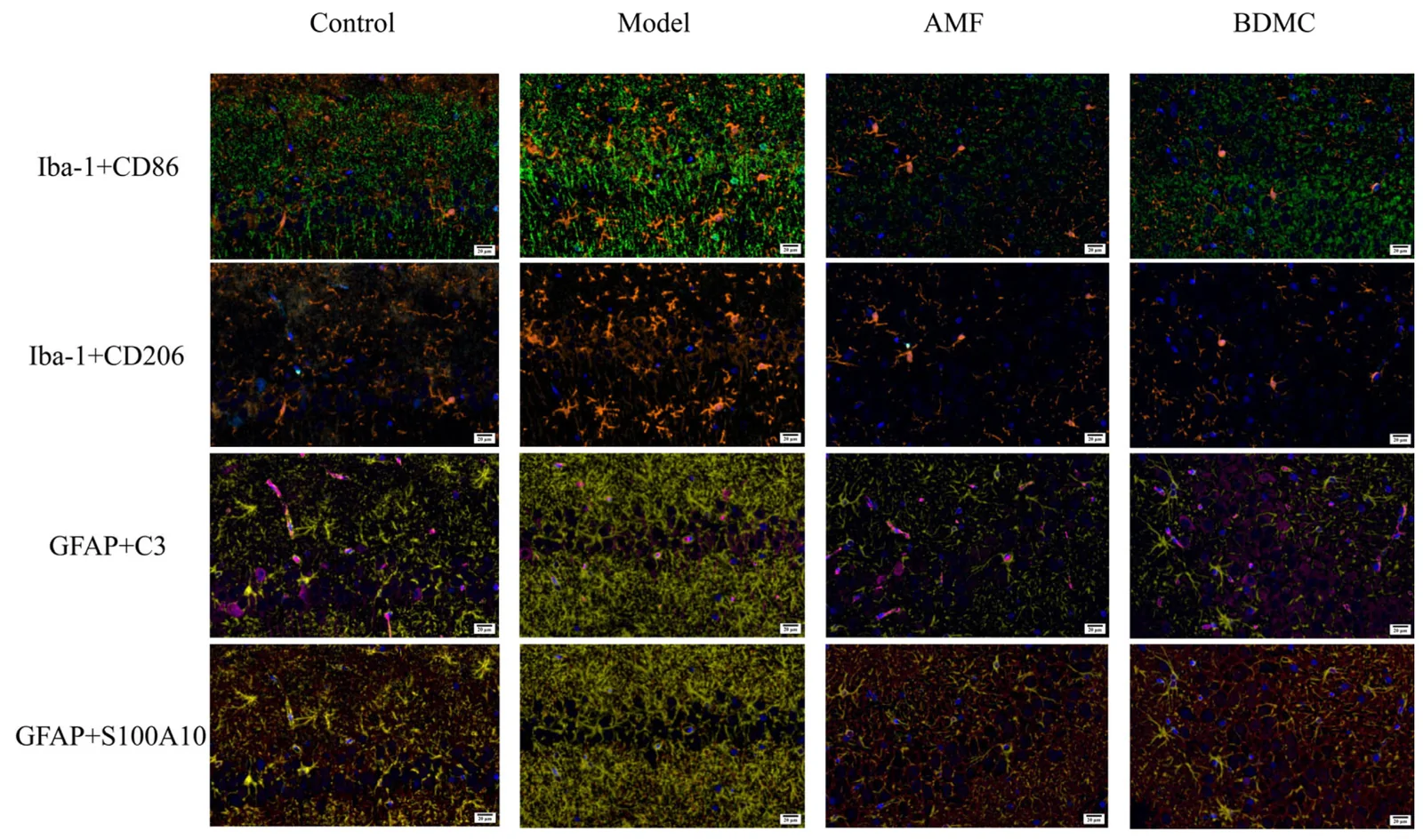
Expression of CD86^+^ microglia/CD206^+^ microglia and C3^+^ astrocyte/S100A10^+^ astrocyte in the hippocampus of RIBI rats (*n* = 3). Scale bar = 20 μm. Representative images were obtained from the hippocampal CA1 astrocyte subfield.

In the hippocampus, the number of CD86^+^ microglia was sharply increased in the Model Group compared with the Control Group (*p* < 0.05), while CD206^+^ microglia showed a mild increase (*p* < 0.05). Both AMF and BDMC interventions markedly reduced the count of CD86^+^ microglia (*p* < 0.05), and the reduction in CD86^+^ microglia was more pronounced in the BDMC Group (*p* < 0.01). The number of CD206^+^ microglia in both treatment groups was lower than that in the Model Group. These results suggest that AMF and BDMC can inhibit CD86^+^ polarization of hippocampal microglia. See Figure 8B and Figure 9 for details.

**Figure 10:**
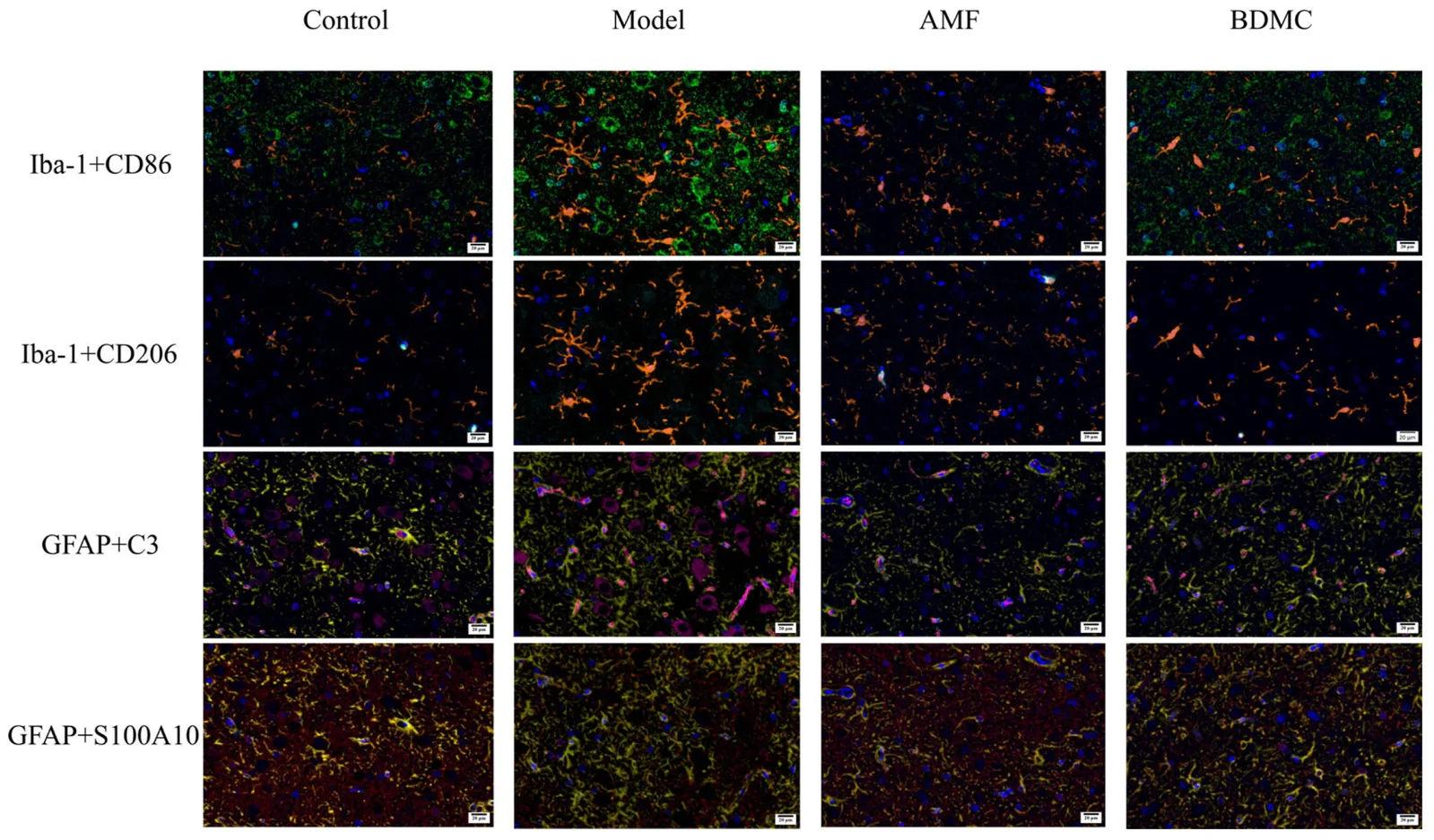
Expression of CD86^+^ microglia/CD206^+^ microglia and C3^+^ astrocyte/S100A10^+^ astrocyte in the thalamus of RIBI rats (*n* = 3). Scale bar = 20 μm. Representative images were obtained from the VPL of the thalamus.

In the thalamus, the numbers of both CD86^+^ microglia and CD206^+^ microglia were substantially elevated in the Model Group compared with the Control Group (*p* < 0.01). AMF intervention led to a significant decrease in CD86^+^ microglia count (*p* < 0.05), accompanied by a concomitant reduction in CD206^+^ microglia. In the BDMC Group, CD86^+^ microglia were markedly decreased (*p* < 0.01), and CD206^+^ microglia levels were obviously decreased relative to the Model Group. These results indicate that AMF and BDMC can effectively suppress CD86^+^ microglia polarization of thalamic microglia. See Figure 8C and Figure 10 for details.

#### 3.7.2. Astrocyte Polarization

In the cerebral cortex, the number of pro-inflammatory C3^+^ astrocytes was significantly increased in the Model Group relative to the Control Group (*p* < 0.01), whereas the number of anti-inflammatory S100A10^+^ astrocytes was substantially decreased (*p* < 0.05). AMF intervention reduced C3^+^ astrocytes and induced a mild recovery of S100A10^+^ astrocyte (*p* < 0.05). BDMC markedly inhibited the excessive activation of C3^+^ astrocyte (*p* < 0.01) and simultaneously elevated the count of S100A10^+^ astrocyte-state. See Figure 7 and Figure 8D for details.

In the hippocampus, the number of C3^+^ astrocyte was significantly increased in the Model Group compared with the Control Group (*p* < 0.05), while the number of S100A10^+^ astrocyte was markedly decreased (*p* < 0.01). Both AMF and BDMC interventions reduced the abundance of C3^+^ astrocyte (*p* < 0.05) and up-regulated S100A10^+^ astrocyte (*p* < 0.05). The promoting effect of BDMC on S100A10^+^ astrocyte was more prominent (*p* < 0.05). See Figure 8E and Figure 9 for details.

In the thalamus, substantial proliferation of C3^+^ astrocyte was observed in the Model Group compared with the Control Group (*p* < 0.05), accompanied by a marked reduction in S100A10^+^ astrocyte (*p* < 0.01). Following AMF intervention, C3^+^ astrocyte decreased while S100A10^+^ astrocyte showed a slight recovery. BDMC significantly reduced the number of C3^+^ astrocyte and increased S100A10^+^ astrocyte count (*p* < 0.01), thereby reversing the imbalance of astrocyte polarization. See Figure 8F and Figure 10 for details.

### 3.8. Interventional Effects of BDMC on the Proportion of Microglial and Astrocytic States

In comparison with the Control Group, the proportion of homeostatic microglia a decreased, the percentage of pro-inflammatory CD86^+^ microglia state significantly increased, and the proportion of anti-inflammatory CD206^+^ microglia state slightly rose in all three brain regions of the Model Group. After AMF and BDMC interventions, the proportion of homeostatic microglia recovered, the percentage of CD86^+^ microglia state markedly declined, and the proportion of CD206^+^ microglia state returned to a lower level. BDMC exhibited superior efficacy in ameliorating the CD86^+^ microglia/CD206^+^ microglia polarization imbalance. See Figure 8G–I for details.

Compared with the Control Group, the proportion of homeostatic astrocyte resting astrocytes decreased, the percentage of pro-inflammatory C3^+^ astrocyte state increased significantly, and the proportion of anti-inflammatory S100A10^+^ astrocyte state declined in all three brain regions of the Model Group. Following AMF and BDMC interventions, the proportion of homeostatic astrocyte was restored, the percentage of C3^+^ astrocyte state was markedly reduced, and the proportion of S100A10^+^ astrocyte state was elevated. These findings suggest that both agents can inhibit the polarization of astrocytes toward the injury-associated state, with BDMC exerting a more pronounced regulatory effect. See Figure 8J–L for details.

## 4. Discussion

Previous studies have demonstrated that radiation-induced brain injury is frequently accompanied by prominent oxidative stress [21]. In general, chronic oxidative stress is regarded as a consequence of the inflammatory response. Immune cells and activated microglia can elevate oxidative stress levels. In addition, radiation-activated microglia may further promote astrogliosis, while cranial irradiation can also directly induce astrocyte activation, which contributes to radiation-induced edema [14]. However, direct irradiation of astrocytes in vitro barely induces gliosis, and the response is predominantly mediated via microglia-dependent pathways [22]. Superoxide dismutase (SOD), catalase (CAT), and glutathione peroxidase (GSH-Px) are key endogenous peroxide-decomposing enzymes, whereas malondialdehyde (MDA) is a product of lipid peroxidation. Therefore, SOD, MDA, GSH-Px, and CAT are frequently used to evaluate the extent of oxidative stress [23]. In the present study, we found that BDMC effectively ameliorated body-weight loss and cerebral edema in RIBI rats. Meanwhile, BDMC alleviated brain injury in RIBI rats, as evidenced by reduced neuronal necrosis and edema. Regarding respect to oxidative stress status, markedly decreased levels of SOD, CAT, and GSH-Px, together with elevated levels of the stress metabolite MDA, were observed in the brain tissues of RIBI rats, indicating that irradiation aggravated cerebral oxidative stress in rats. After intraperitoneal injection of BDMC, the suppressed expression of SOD, CAT and GSH-Px was restored, accompanied by reduced MDA production. Collectively, these results suggest that BDMC exerts cytoprotective effects in RIBI rats by up-regulating antioxidant enzymes, mitigating cerebral oxidative stress, and lowering the accumulation of peroxidation products.

Microglia are the resident immune cells of the CNS and play a critical role in RIBI. When the brain is exposed to IR, soluble factors on the neuronal surface that normally restrain microglial reactivity are disrupted [24]. Microglial polarization states may also participate in the brain’s response to IR. Post-irradiation microglial reactivity triggers the release of inflammatory mediators and mediates crosstalk among neurons, astrocytes, pericytes and infiltrating peripheral immune cells, thereby amplifying neuroinflammation, blood–brain barrier disruption and progressive RIBI [25]. In addition, different states may reflect distinct physiological functions of microglia after IR exposure. Reactive microglia, characterized by different marker expression profiles (e.g., CD86^+^ and CD206^+^), produce distinct inflammatory mediators and thereby contribute to diverse physiological processes. Following high-dose radiation exposure, microglia show increased abundance of CD86^+^ cells within the population. These cells exhibit amoeboid morphology, enhanced phagocytic capacity, and secrete multiple pro-inflammatory mediators, including interleukin-1β (IL-1β), interleukin-6 (IL-6), reactive oxygen species (ROS), and tumor necrosis factor-α (TNF-α). Furthermore, these cells exert distinct inhibitory effects on the generation, differentiation, and survival of neural progenitors [26]. Pharmacologically inducing the phenotypic switch of microglia from CD86^+^ microglia toward CD206^+^ microglia to alleviate tissue damage in RIBI models may represent a promising therapeutic strategy for RIBI [27].

In the present study, we observed that BDMC treatment effectively inhibited radiation-induced up-regulation of Iba-1 in the cerebral cortex, hippocampus, and thalamus, reduced microglial activation, and improved the cerebral microenvironment in the RIBI model. Robust up-regulation of CD206^+^ microglia was noted in the Model group. This may be attributed to the massive production of inflammatory factors upon CNS irradiation, which triggers inflammatory responses and further up-regulates CD206^+^ microglia to counteract inflammation. This observation is consistent with our previous findings [28]. To further explore the modulatory effect of BDMC on microglial polarization, we performed in situ labeling for the CD86^+^ microglial marker CD86 and the CD206^+^ microglial marker CD206 on brain sections. We found that the number of Iba-1^+^CD86^+^ and Iba-1^+^/CD206^+^ double-positive cells was markedly decreased in the cerebral cortex of the BDMC group. In the hippocampus and thalamus, the count of Iba-1^+^/CD86^+^ double-positive cells was also significantly reduced in the BDMC group. These results indicated that BDMC effectively reduced the abundance of CD86^+^ microglia in the RIBI model. However, its influence on CD206^+^ microglial counts was inconsistent, with only a modest reduction observed in the cerebral cortex. This discrepancy may be attributable to the limited sample size, which could account for the non-significant statistical findings.

As the most abundant cell type in the central nervous system, astrocytes undergo remarkable changes in state, function, and gene expression upon harmful stimuli and neural injury, a process termed reactive astrogliosis [29]. Reactive astrocytes can support neuronal activity and survival through the release of signaling molecules. Astrocytes facilitate neuronal communication and participate in neurotransmitter recycling. They also contribute to brain protection against trauma, infection, and neurodegeneration, maintaining cerebral homeostasis and function [30]. Several research groups have proposed that neural injury can induce two distinct states of reactive astrocytes, namely C3^+^ astrocytes and S100A10^+^ reactive astrocytes [31]. Interestingly, studies have reported that in rat hippocampal inflammatory models and U87-MG astrocyte cultures, γ-ray irradiation tends to induce pro-inflammatory and damaging states, accompanied by caspase-8/3 activation and elevated expression of GFAP, C3 and Pentraxin 3 (PTX3). In contrast, proton irradiation attenuates glial reactivity, increases glutamine synthetase expression, and upregulates miR-92a-3p, which in turn reduces GFAP and IL-6 levels. These findings indicate that different radiation qualities can evoke distinct patterns of astrocyte reactivity and inflammatory responses [32]. C3^+^ astrocytes can trigger rapid death of neurons and oligodendrocytes, whereas neuroprotective S100A10^+^ astrocytes promote neuronal survival and tissue repair [33]. Activated microglia drive the transformation of naive astrocytes toward the C3^+^ astrocyte state by secreting IL-1, TNF-α, and complement component 1q (C1q) [31]. Studies on low-dose radiation simulating space-relevant radiation have also revealed that astrocytes exacerbate blood–brain barrier permeability in the acute phase after irradiation. By contrast, in the sub-acute phase, astrocytes shift toward a more protective state by reducing oxidative stress and the secretion of pro-inflammatory cytokines and chemokines [34].

In the present study, we observed that GFAP expression was suppressed in the cerebral cortex, hippocampus, and thalamus of the BDMC group, indicating that BDMC effectively inhibited astrocytic activation and modulated the proportion of activated glial cells in the RIBI model. To further investigate the regulatory effect of BDMC on astrocyte polarization, we performed in situ labeling for the C3^+^ astrocyte marker C3 and the S100A10^+^ astrocyte marker S100A10 on brain sections. We found that the number of GFAP^+^C3^+^ double-positive cells was markedly decreased in the cerebral cortex and hippocampus in the BDMC group, whereas the count of GFAP^+^/S100A10^+^ double-positive cells was significantly increased in these two brain regions. A significant up-regulation of GFAP^+^/S100A10^+^ double-positive cells was also detected in the thalamus. Collectively, these findings demonstrate that BDMC effectively inhibits polarization toward the C3^+^ astrocyte state and promotes polarization toward the S100A10^+^ astrocyte state in astrocytes under RIBI conditions.

The present study confirms that prominent dysregulation of glial polarization occurs in three key brain regions, namely the cerebral cortex, hippocampus, and thalamus, after model establishment. As the resident innate immune cells of the central nervous system, microglia undergo robust polarization toward the pro-inflammatory CD86^+^ microglia state upon injurious stimulation, accompanied by a marked increase in the number and proportion of CD86^+^ microglia, together with a concurrent reduction in the proportions of resting homeostatic microglia and anti-inflammatory reparative CD206^+^ microglia. Meanwhile, astrocytes exhibit pathological activation, with a substantial elevation in the number and proportion of pro-damaging C3^+^ astrocyte states and a notable decrease in protective S100A10^+^ astrocyte states. The cerebral cortex serves as the core brain region governing perception, motor function, higher-order cognition, and information integration. Its widely distributed neuronal and synaptic networks are highly susceptible to inflammatory microenvironments. Robust proliferation of CD86^+^ microglia and C3^+^ astrocytes can be observed in the cerebral cortex of model animals. Imbalanced glial polarization directly disrupts cortical synaptic connections, suppresses neuronal electrical activity, and consequently triggers abnormalities in neural perception and behavioral regulation [35]. The hippocampus, the core brain region for learning and memory, exhibits the most pronounced increase in CD86^+^ microglia and C3^+^ astrocytes. This finding is highly consistent with the pathological hallmarks of cognitive decline and neuronal apoptosis following radiation-induced brain injury [36]. The thalamus participates in neural signal relay, whereas the cerebral cortex mediates higher-order neural regulation. Imbalanced glial polarization in both regions further exacerbates neural circuit dysfunction [37]. CD86^+^ microglia secrete abundant pro-inflammatory factors such as TNF-α and IL-1β, while C3^+^ astrocytes release neurotoxic mediators. Their synergistic effects create a chronic inflammatory microenvironment that continuously damages neurons, forming a vicious cycle: inflammatory responses, neuronal injury, and subsequent escalation of glial reactivity. This may represent an important pathological mechanism contributing to neurological deficits in these model rats.

Following intervention with AMF and BDMC, the numbers of pro-inflammatory and damaging CD86^+^ microglia and C3^+^ astrocytes were markedly reduced in all three brain regions, accompanied by recovery in the population of protective CD206^+^ microglia and S100A10^+^ astrocyte states. Subtype proportion data further corroborated that both agents increased the percentage of resting homeostatic microglia and homeostatic astrocyte glial cells while decreasing the proportion of CD86^+^ microglia and C3^+^ astrocyte states, thereby effectively correcting the aberrant glial polarization shift. CD206^+^ microglia secrete anti-inflammatory factors including IL-10 and TGF-β, clear damaged cellular debris, and facilitate tissue repair. S100A10^+^ astrocytes preserve blood–brain barrier integrity, provide nutritional support for neurons, and suppress oxidative stress. By modulating glial polarization states, both agents restrain excessive central inflammatory responses and re-establish immune homeostasis within neural tissue, consequently alleviating glia-mediated secondary brain injury. Comparison of therapeutic efficacy between the two treatment groups revealed that BDMC exhibited superior effects in suppressing CD86^+^ microglia/C3^+^ astrocyte states and elevating CD206^+^ microglia/S100A10^+^ astrocyte states relative to AMF. These findings suggest that BDMC possesses greater neuroprotective potential in regulating glial polarization and mitigating neuroinflammation.

We consider the attenuation of oxidative stress to be one of the key mechanisms underlying the suppressive effects of BDMC and AMF on glial activation. Cranial irradiation directly induces excessive ROS production, triggering lipid peroxidation and oxidative damage to neurons and glial cells. In this study, irradiation markedly elevated MDA, a well-recognized marker of lipid peroxidation, and reduced the activities of antioxidant enzymes SOD, CAT and GSH-Px. Disruption of this antioxidant defense system generates an oxidative microenvironment that enhances microglial and astrocytic reactivity. Notably, both AMF and BDMC reversed these alterations: they restored SOD, CAT and GSH-Px activities and lowered MDA accumulation, reflecting improved free radical scavenging capacity and alleviated oxidative load. Since oxidative stress is a well-documented trigger driving glial pro-inflammatory transformation, the recovery of redox balance by BDMC and AMF may account for the decreased pro-inflammatory state of microglia and astrocytes. Taken together, we propose that the inhibition of oxidative stress, demonstrated by normalized SOD, CAT, GSH-Px and MDA levels, represents one potential mechanism by which BDMC and AMF mitigate neuroinflammation and cranial radiation-induced brain injury.

## 5. Conclusions

In this study, an RIBI model was established using SD rats. Body-weight monitoring, brain edema rate measurement, oxidative-stress assessment, HE staining, and multiplex immunohistochemistry (mIHC) were applied to systematically evaluate the intervention effect of BDMC on radiation-induced brain injury and glial polarization in RIBI model rats. The results demonstrate that the neuroprotective mechanism of BDMC against RIBI may involve the regulation of CD86^+^ microglia/CD206^+^ microglia and C3^+^ astrocyte/S100A10^+^ astrocyte polarization balance. Specifically, BDMC reduces the number of pro-inflammatory CD86^+^ microglia and C3^+^ astrocytes across multiple brain regions, increases protective CD206^+^ microglia and S100A10^+^ astrocyte states, reverses the disordered proportions of glial states, restores neuroinflammatory homeostasis, and alleviates cerebral oxidative stress.

## Figures and Tables

**Table 1:** Semi-quantitative histopathological scoring of different brain regions (mean ± SD).

	Control	Model	AMF	BDMC
Cerebral cortex	0	4.33 ± 0.47 ##	0.67 ± 0.47 **	1 ± 0.82 **
Hippocampus	0	3 ± 0.82 #	0.67 ± 0.47 *	1 *
Thalamus	0	3 ± 0.82 #	0.47 *	1 ± 0.47 *

Notes: Data in this figure were analyzed by one-way ANOVA with LSD post hoc test. The significance level was set at 0.05. * *p* < 0.05, ** *p* < 0.01 vs. Model group; # *p* < 0.05, ## *p* < 0.01 vs. Control group.

## Data Availability

The original contributions presented in this study are included in the article. Further inquiries can be directed to the corresponding authors.
